# Evaluation of the toll-like receptor 6 Ser249Pro polymorphism in patients with asthma, atopic dermatitis and chronic obstructive pulmonary disease

**DOI:** 10.1186/1471-2350-6-34

**Published:** 2005-09-28

**Authors:** Sabine Hoffjan, Susanne Stemmler, Qumar Parwez, Elisabeth Petrasch-Parwez, Umut Arinir, Gernot Rohde, Karin Reinitz-Rademacher, Gerhard Schultze-Werninghaus, Albrecht Bufe, Jörg T Epplen

**Affiliations:** 1 Department of Human Genetics, Ruhr-University Bochum, Germany; 2 Private medical practice, Gladbeck, Germany; 3 Department of Neuroanatomy and Molecular Brain Research, Ruhr-University Bochum, Germany; 4 Department of Internal Medicine lll, Pneumology, Allergology and Sleep Medicine, Ruhr-University Bochum, Germany; 5 Department of Experimental Pneumology, Ruhr-University Bochum, Germany

## Abstract

**Background:**

For allergic disorders, the increasing prevalence over the past decade has been attributed in part to the lack of microbial burden in developed countries ('hygiene hypothesis'). Variation in genes encoding toll-like receptors (TLRs) as the receptor system for the first innate immune response to microbial stimuli has been implicated in various inflammatory diseases. We evaluated here the role of a coding variation, Ser249Pro, in the *TLR6 *gene in the pathogenesis of asthma, atopic dermatitis (AD) and chronic obstructive pulmonary disease (COPD).

**Methods:**

Genotyping of the Ser249Pro polymorphism in 68 unrelated adult patients and 132 unrelated children with asthma, 185 unrelated patients with COPD, 295 unrelated individuals with AD and 212 healthy control subjects was performed by restriction enzyme digestion.

**Results:**

We found a weak association of the 249Ser allele with childhood asthma (p = 0.03). Yet, significance was lost after Bonferroni correction. No association was evident for AD or COPD.

**Conclusion:**

Variation in *TLR6 *might play a role in the pathogenesis of childhood asthma.

## Background

Asthma, atopic dermatitis (AD) and chronic obstructive pulmonary disease (COPD) are common chronic inflammatory diseases with prevalence rates between 5 and 15%, making them major public-health problems worldwide [1,2]. For all three diseases, a multifactorial background has been suggested with genetic as well as environmental factors influencing disease susceptibility [3-5]. There is now strong evidence that exposure to microbial products in early childhood plays an important role in the postnatal maturation of the immune system and that the increase in the prevalence of allergic diseases over the last few decades might be in part due to the decreased microbial burden in industrialized countries (the so-called hygiene hypothesis) [6,7]. The receptor system that constitutes the first, innate immune response to microbial stimuli consists of the family of toll-like receptors (TLRs), highly conserved receptor complexes that recognize pathogen-associated molecular patterns (PAMPs) [8]. Currently, 10 different TLRs are known each of which recognizes a different spectrum of PAMPs. As activators of cytokine production in response to infections, TLRs are believed to be involved in the establishment of T helper (Th)1 immune responses in early life, counter-balancing the Th2-dominated cytokine spectrum at birth [9]. Genetic variation in these receptors could thus influence susceptibility for both Th1- (*e.g*., autoimmune) and Th2-mediated (*e.g*., allergic) diseases [9].

While several studies have evaluated the role of variation in *TLR4*, the receptor for endotoxin, for allergic diseases [10-12], little is known about the role of *TLR6 *variation so far. TLR6 forms heterodimers with TLR2 for recognition of bacterial lipopeptides [13]. Expression of TLR6 has been demonstrated in human mast cells [14] which play an important role in allergic diseases [15]. Interestingly, activation of mast cells *via*, both, TLR4 and TLR2/TLR6 resulted in additive effects on cytokine production [16]. Thus, *TLR6 *appears as interesting candidate gene for allergic disorders. The *TLR6 *gene is located on chromosome 4p13 and it comprises a single exon encoding a 796 amino-acid polypeptide. Recently, a common coding polymorphism was identified in this gene that leads to an exchange from serine to proline at position 249 in the extracellular domain of the TLR6 protein [17]. In preliminary analyses, the T allele (249Ser) was associated with protection from asthma in African American samples, while the same trend was not significant for European Americans [17]. Although the association reported in this study is intriguing considering the hygiene hypothesis, a replication of this finding for asthma in a different sample has not yet been accomplished. We further speculated that *TLR6 *coding variation might also play a role in the pathogenesis of COPD since it is – like asthma – a common chronic lung disease characterized by chronic airway inflammation and airway hyper-responsiveness [1]. In addition, there is growing evidence that at least part of the genetic background might be common among COPD and asthma [18]. We therefore studied the *TLR6 *Ser249Pro polymorphism in four different German cohorts: adult asthmatics, pediatric asthmatics, patients with COPD and patients with AD in comparison with matched German controls.

## Methods

### Subjects

185 unrelated adult patients with COPD and 68 unrelated adult patients with asthma were recruited while hospitalized at the Bergmannsheil clinics, Ruhr-University Bochum, Germany, and 132 unrelated children with asthma in the Pediatric Pneumology Studycenter (PPS) located at the same place. The COPD diagnosis was based on the classification of the Global Initiative for Chronic Obstructive Lung Disease (GOLD) criteria [19]. Patients included in the study had at least a forced expiratory volume in one second (FEV_1_) of < 80% of predicted and a FEV_1_/FVC (forced vital capacity) ratio of < 70% of predicted, classified as moderate to severe COPD (GOLD stages II-IV). Adult asthmatics had a doctor's diagnosis of asthma according to the standards of the American Thoracic Society [20]. The exclusion criteria were α1-antitrypsin deficiency, dyspnea of other origin (including cardiovascular disorders, pneumonia, interstitial lung disease, pleural disease, upper airways obstruction, neuromuscular disease and anaemia) and bronchial carcinoma. For the asthmatic children, the asthma diagnosis was based on the ISAAC questionnaire [21]. Disease severity was assessed by a symptom score recently validated [22]. 294 unrelated patients with AD were recruited by a consultant specialist for AD (QP, Gladbeck, Germany), including 175 children and 119 adults. The AD diagnosis was based on the presence of clinical features, including purities, eczema with age-dependent differences in location, xerosis and chronic or relapsing dermatitis. In addition, all investigated AD patients had a positive family history for atopic diseases.

212 control samples from adults without known allergies, asthma or AD were collected in the same private practice as the AD patients. We specifically chose to use non-allergic adults as controls because for diseases as frequent as asthma and AD, the risk remains very high for asymptomatic children to develop an allergic disease during childhood or even adulthood [23,24]. The control subjects underwent clinical examination in order to exclude symptoms of asthma, AD or COPD, had no self-reported allergies or allergic symptoms and no first degree relatives with known allergic diseases. Lung function tests or IgE measures were not performed for the controls. All patient and control subjects were Caucasians of German origin. Informed consent was obtained from all subjects. The study was approved by the Ethics Committee of the University of Bochum and the Declaration of Helsiniki protocols were followed. DNA was extracted from EDTA anti-coagulated peripheral blood by using a standard salting-out method [25].

### Genotyping

Genotyping of the *TLR6 *Ser249Pro polymorphism was performed by polymerase chain reaction (PCR) with subsequent restriction enzyme digestion. PCR reactions were performed in a total volume of 10 μl, containing 50 ng DNA, 200 mmol of each dNTP, 3 mmol MgCl_2_, 10 pmol of each primer (forward: GCATTTCCAAGTCGTTTCTATGT; reverse: GCAAAAACCCTTCACCTTGTT), and 0.4 U Taq polymerase (Genecraft, Münster, Germany). Thermal cycling was performed (Biometra, Göttingen, Germany). After two initial cycles at 6°C and 3°C above the annealing temperature, 27 cycles of 95°C (30 sec), 57°C (60 sec) and 72°C (60 sec) were run. The PCR product was digested with Ava II (0.01 U/ng DNA) at 37°C for three hours. The fragments were subsequently separated on 2% agarose gels in 1 × TBE buffer (30 min, 200 V) and visualized using ethidium bromide staining.

### Statistical analyses

Genotype and allele frequencies were ascertained by direct counting and subsequently analyzed according to the χ^2 ^method. Deviations from Hardy-Weinberg equilibrium were evaluated using the FINETTI program. P < 0.05 was considered to be significant. Power calculations were performed using the *Power and sample size program *[26].

## Results and discussion

Clinical data concerning the four patient groups are summarized in table 1. Since the nucleotide substitution from C to T at position 744 in the *TLR6 *gene results in the creation of a new restriction site, the Ser249Pro polymorphism was genotyped by restriction enzyme digestion. Genotype frequencies for case and control groups are shown in table 2. The frequencies in all five groups were in Hardy-Weinberg equilibrium. There was a significant association between the T allele (249Ser) and childhood asthma (p = 0.03, table 3). Yet, significance was lost after Bonferroni correction. For the other three case groups, no significant association was observed.

Although it seems puzzling to identify the opposite allele being associated with childhood asthma as compared to the one reported by Tantisira et al. [17], there are plausible explanations for this phenomenon. First, the association with childhood asthma reported here is only a weak association that did not withstand Bonferroni correction. Yet, we consider the study to be explorative and hypothesis-generating and find the results intriguing in the light of the emerging role of TLRs in allergic diseases [9]. In addition, the reported association refers to a coding variation that might at least theoretically have functional consequences. It remains largely unknown what sort of genetic variants explain inherited variation in complex traits, but recent evidence suggests that common, non-coding genetic variants will explain at least some of the inherited variation in susceptibility to common disease [27]. Thus, testing a single functional variation would be more likely biased into the direction of a negative result rather than of a positive association.

Second, the previous association of the 249Ser allele with protection from asthma was defined in a sample of African American patients and controls. The minor allele frequency of the Ser249Pro polymorphism in this cohort was 0.08 in asthmatics and 0.19 in controls [17] and thus much lower than in our four German cohorts with a minor allele frequency of on average 0.40 (range 0.35 to 0.49). Yet, the allele frequencies we observed in this study are comparable to the ones for European Americans [28]. It appears possible that the Ser249Pro polymorphism might not be disease-causing by itself, but instead be in linkage disequilibrium with the true disease-causing variation. In this case, different alleles of Ser249Pro could be linked to the relevant allele in different populations (*e.g*., African Americans and Caucasians). Further association studies in these and other populations are needed to answer this question.

Third, environmental factors might play a role in disease susceptibility associated with opposite alleles. One such example has been described for CD14, which associates with TLR4 to form the receptor complex that recognizes lipopolysaccharide (LPS, endotoxin). A single nucleotide polymorphism (SNP) in the *CD14 *promoter at position -159 (C/T) was identified [29]. The -159T allele was associated with high levels of soluble CD14 and decreased total serum IgE in a cohort of children from Tucson, Arizona [29]. Yet, no association of this SNP with allergy or IgE levels was evident in a large German cohort [30]. In the Hutterites, an isolated population from South Dakota, the -159T allele was instead associated with increased risk for atopy [31]. Vercelli recently postulated an intriguing explanation for this phenomenon: she suggested that the level of endotoxin exposure influences the 'switch' from the Th2-biased cytokine profile at birth to a Th1-biased cytokine profile in early childhood, and that endotoxin levels might interact with the *CD14 *genotype to confer either risk to or protection from atopic phenotypes later in life [32]. Thus, environmental factors – and even endotoxin load – might also be responsible for the discrepancy between the findings of Tantisira et al. [17] and our results, concerning the role of the Ser249Pro polymorphism in asthma pathogenesis. Since we did not yet measure endotoxin levels, we are unable to explore this question at the moment.

Association studies of polymorphisms in other *TLR *genes have also revealed somewhat contradictory results. Two missense mutations (Asp299Gly and Thr399Ile) in the gene encoding TLR4 which mediates the biological response to LPS were associated with a decreased response to inhaled endotoxin in humans [33]. Since exposure to LPS in early life has been suggested to exert protective effects on the development of allergic diseases [34-36], the *TLR4 *gene appeared to be an outstanding functional candidate for the susceptibility to asthma and allergic diseases. Yet only one out of four association studies found a direct association of the *TLR4 *Asp299Gly polymorphism with asthma [10]. In three other studies, no differences in the overall risk for asthma between carriers of the wild type and mutant genotypes were obvious [11,12,37]. On the other hand, an impact of this polymorphism on the severity of atopy in asthmatics [11] was reported as well as a modified response to endotoxin [12], indicating gene/environment interplay. Similarly, a polymorphism at position -16934 in the *TLR2 *gene was significantly associated with asthma and atopy in farmers' children but not in children that did not grow up on farms [38], pointing again to a gene/environment interaction. Thus, variation in *TLR *genes appears to have modifying effects on asthma susceptibility.

The fact that we did not find an association of the Ser249Pro polymorphism with adult asthma might be due to the small sample size of adult asthmatics. Yet, in order to achieve a significance level of 0.05 with a statistical power of 80%, a sample size of over 500 patients would have been required. Replication studies in larger cohorts as well as functional studies are clearly needed. To our knowledge, this is the first evaluation of the *TLR6 *Ser249Pro polymorphism in patients with atopic dermatitis, and we did not find association with this allergic skin disease, suggesting that the observed association might be restricted to (childhood) asthma, rather than allergic diseases in general. This finding is not totally unexpected since the results of genome-wide screens indicate that loci linked to AD do not overlap substantially with loci linked to asthma and related phenotypes, but rather with loci for psoriasis, another chronic skin disease [4]. Furthermore, we did not find evidence that *TLR6 *Ser249Pro contributes to susceptibility for COPD.

## Conclusion

We found a weak association between the *TLR6 *Ser249Pro polymorphism and risk for childhood asthma, while no association was evident for AD or COPD. Analysis of the effect of this variation on disease severity and potential gene/environment interactions might be a promising approach since gene/environment interactions have been described for other *TLR *genes. Further studies of the *TLR6 *Ser249Pro polymorphism in ethnically well-defined cohorts as well as functional studies of this variation are warranted to evaluate its role in the pathogenesis of allergic diseases.

## List of abbreviations

AD atopic dermatitis

COPD chronic obstructive pulmonary disease

FEV_1 _forced exspiratory volume in the first second

FVC forced vital capacity

LPS lipopolysaccharide

PAMP pathogen-associated molecular pattern

PCR polymerase chain reaction

TLR toll-like receptor

## Competing interests

The author(s) declare that they have no competing interests.

## Authors' contributions

SH was in charge of the design and coordination of the atopic dermatitis association study, performed the statistical analysis and drafted the manuscript. SS coordinated the asthma and COPD association studies. EPP participated in the design and coordination of the AD study. QP, UA, GR and KRR participated in the recruitement of patients and clinical data collection. GSW, AB and JTE participated in the design and coordination of the whole study and helped to draft the manuscript.

## Pre-publication history

The pre-publication history for this paper can be accessed here:



## Figures and Tables

**Table 1:** Clinical data of the asthma, AD and COPD cohorts as well as the control group

	Adult patients with asthma	Children with asthma	AD	COPD	Healthy controls
number of subjects	68	132	294	182	212
gender: f/m	36/32	52/80	180/114	62/120	131/81
age (years) [median(range)]	38 (18–68)	9.5 (2–15)	12 (0.5–72)	68 (33–81)	59 (22–87)
FEV1 (%pred.) (mean ± STD)	73 ± 25	97 ± 18	--	47 ± 19	--
FEV1/FVC ratio (mean ± STD)	65 ± 16	98 ± 15	--	49 ± 15	--
pack years [median(range)]	2 (0–44)	--	--	30 (0–200)	--

**Table 2:** Genotype frequencies of the *TLR6 *Ser249Pro polymorphism in the case and control groups.

*TLR6 *Ser249Pro	Controls	Asthma adults	Asthma children	AD	COPD
	N = 212	N = 68	N = 132	N = 294	N = 185
					
Pro/Pro	75	28	32	108	76
Pro/Ser	104	32	72	138	80
Ser/Ser	33	8	28	48	29

p-value	-	n.s.	0.07	n.s.	n.s.

**Table 3:** Allele frequencies of the *TLR6 *Ser249Pro polymorphism in asthma children *vs*. controls.

	Asthma children N = 132	Controls N = 212
Pro	136 (51.5%)	254 (59.9%)
Ser	128 (48.5%)	170 (40.1%)
	p = 0.03	

## References

[B1] Skrepnek GH, Skrepnek SV (2004). Epidemiology, clinical and economic burden, and natural history of chronic obstructive pulmonary disease and asthma. Am J Manag Care.

[B2] Leung DY, Bieber T (2003). Atopic dermatitis. Lancet.

[B3] Hoffjan S, Ober C (2002). Present status on the genetic studies of asthma. Curr Opin Immunol.

[B4] Bowcock AM, Cookson WO (2004). The genetics of psoriasis, psoriatic arthritis and atopic dermatitis. Hum Mol Genet.

[B5] Molfino NA (2004). Genetics of COPD. Chest.

[B6] Martinez FD (2001). The coming-of-age of the hygiene hypothesis. Respir Res.

[B7] Weiss ST (2002). Eat dirt--the hygiene hypothesis and allergic diseases. N Engl J Med.

[B8] Janssens S, Beyaert R (2003). Role of Toll-like receptors in pathogen recognition. Clin Microbiol Rev.

[B9] Cook DN, Pisetsky DS, Schwartz DA (2004). Toll-like receptors in the pathogenesis of human disease. Nat Immunol.

[B10] Fageras Bottcher M, Hmani-Aifa M, Lindstrom A, Jenmalm MC, Mai XM, Nilsson L, Zdolsek HA, Bjorksten B, Soderkvist P, Vaarala O (2004). A TLR4 polymorphism is associated with asthma and reduced lipopolysaccharide-induced interleukin-12(p70) responses in Swedish children. J Allergy Clin Immunol.

[B11] Yang IA, Barton SJ, Rorke S, Cakebread JA, Keith TP, Clough JB, Holgate ST, Holloway JW (2004). Toll-like receptor 4 polymorphism and severity of atopy in asthmatics. Genes Immun.

[B12] Werner M, Topp R, Wimmer K, Richter K, Bischof W, Wjst M, Heinrich J (2003). TLR4 gene variants modify endotoxin effects on asthma. J Allergy Clin Immunol.

[B13] Takeda K, Akira S (2005). Toll-like receptors in innate immunity. Int Immunol.

[B14] McCurdy JD, Olynych TJ, Maher LH, Marshall JS (2003). Cutting edge: distinct Toll-like receptor 2 activators selectively induce different classes of mediator production from human mast cells. J Immunol.

[B15] Boyce JA (2003). The role of mast cells in asthma. Prostaglandins Leukot Essent Fatty Acids.

[B16] Hume DA, Underhill DM, Sweet MJ, Ozinsky AO, Liew FY, Aderem A (2001). Macrophages exposed continuously to lipopolysaccharide and other agonists that act via toll-like receptors exhibit a sustained and additive activation state. BMC Immunol.

[B17] Tantisira K, Klimecki WT, Lazarus R, Palmer LJ, Raby BA, Kwiatkowski DJ, Silverman E, Vercelli D, Martinez FD, Weiss ST (2004). Toll-like receptor 6 gene (TLR6): single-nucleotide polymorphism frequencies and preliminary association with the diagnosis of asthma. Genes Immun.

[B18] Meyers DA, Larj MJ, Lange L (2004). Genetics of asthma and COPD. Similar results for different phenotypes. Chest.

[B19] Hurd S, Pauwels R (2002). Global Initiative for Chronic Obstructive Lung Diseases (GOLD). Pulm Pharmacol Ther.

[B20] (1987). Standards for the diagnosis and care of patients with chronic obstructive pulmonary disease (COPD) and asthma. This official statement of the American Thoracic Society was adopted by the ATS Board of Directors, November 1986. Am Rev Respir Dis.

[B21] Asher MI, Keil U, Anderson HR, Beasley R, Crane J, Martinez F, Mitchell EA, Pearce N, Sibbald B, Stewart AW (1995). International Study of Asthma and Allergies in Childhood (ISAAC): rationale and methods. Eur Respir J.

[B22] Bufe A, Ziegler-Kirbach E, Stoeckmann E, Heidemann P, Gehlhar K, Holland-Letz T, Braun W (2004). Efficacy of sublingual swallow immunotherapy in children with severe grass pollen allergic symptoms: a double-blind placebo-controlled study. Allergy.

[B23] Bel EH (2004). Clinical phenotypes of asthma. Curr Opin Pulm Med.

[B24] De Marco R, Locatelli F, Cerveri I, Bugiani M, Marinoni A, Giammanco G (2002). Incidence and remission of asthma: a retrospective study on the natural history of asthma in Italy. J Allergy Clin Immunol.

[B25] Miller SA, Dykes DD, Polesky HF (1988). A simple salting out procedure for extracting DNA from human nucleated cells. Nucleic Acids Res.

[B26] The Vanderbilt Medical Center Power and Sample Size Calculation. http://www.mc.vanderbilt.edu/prevmed/ps.

[B27] Newton-Cheh C, Hirschhorn JN (2005). Genetic association studies of complex traits: design and analysis issues. Mutat Res.

[B28] Innate Immunity in Heart, Lung and Blood disease. http://www.innateimmunity.net.

[B29] Baldini M, Lohman IC, Halonen M, Erickson RP, Holt PG, Martinez FD (1999). A Polymorphism* in the 5' flanking region of the CD14 gene is associated with circulating soluble CD14 levels and with total serum immunoglobulin E. Am J Respir Cell Mol Biol.

[B30] Sengler C, Haider A, Sommerfeld C, Lau S, Baldini M, Martinez F, Wahn U, Nickel R (2003). Evaluation of the CD14 C-159 T polymorphism in the German Multicenter Allergy Study cohort. Clin Exp Allergy.

[B31] Ober C, Tsalenko A, Parry R, Cox NJ (2000). A second-generation genomewide screen for asthma-susceptibility alleles in a founder population. Am J Hum Genet.

[B32] Vercelli D (2003). Learning from discrepancies: CD14 polymorphisms, atopy and the endotoxin switch. Clin Exp Allergy.

[B33] Arbour NC, Lorenz E, Schutte BC, Zabner J, Kline JN, Jones M, Frees K, Watt JL, Schwartz DA (2000). TLR4 mutations are associated with endotoxin hyporesponsiveness in humans. Nat Genet.

[B34] Braun-Fahrlander C (2001). The role of the farm environment and animal contact for the development of asthma and allergies. Clin Exp Allergy.

[B35] Gehring U, Bischof W, Fahlbusch B, Wichmann HE, Heinrich J (2002). House dust endotoxin and allergic sensitization in children. Am J Respir Crit Care Med.

[B36] Gehring U, Bischof W, Schlenvoigt G, Richter K, Fahlbusch B, Wichmann HE, Heinrich J (2004). Exposure to house dust endotoxin and allergic sensitization in adults. Allergy.

[B37] Raby BA, Klimecki WT, Laprise C, Renaud Y, Faith J, Lemire M, Greenwood C, Weiland KM, Lange C, Palmer LJ, Lazarus R, Vercelli D, Kwiatkowski DJ, Silverman EK, Martinez FD, Hudson TJ, Weiss ST (2002). Polymorphisms in toll-like receptor 4 are not associated with asthma or atopy-related phenotypes. Am J Respir Crit Care Med.

[B38] Eder W, Klimecki W, Yu L, von Mutius E, Riedler J, Braun-Fahrlander C, Nowak D, Martinez FD (2004). Toll-like receptor 2 as a major gene for asthma in children of European farmers. J Allergy Clin Immunol.

